# Structural and Functional Characterization of RecG Helicase under Dilute and Molecular Crowding Conditions

**DOI:** 10.1155/2012/392039

**Published:** 2012-08-08

**Authors:** Sarika Saxena, Satoru Nagatoishi, Daisuke Miyoshi, Naoki Sugimoto

**Affiliations:** ^1^Frontier Institute for Biomolecular Engineering Research (FIBER), Konan University, 7-1-20 Minatojima-Minamimachi, Chuo-ku, Kobe 650-0047, Japan; ^2^Amity Institute of Biotechnology, Amity University Uttar Pradesh, Sector-125, Expressway Highway, Noida 201303, India; ^3^Department of Nanobiochemistry, Faculty of Frontiers of Innovative Research in Science and Technology (FIRST), Konan University, 7-1-20 Minatojima-Minamimachi, Chuo-ku, Kobe 650-0047, Japan

## Abstract

In an ATP-dependent reaction, the *Escherichia coli* RecG helicase unwinds DNA junctions *in vitro*. We present evidence of a unique protein conformational change in the RecG helicase from an **α**-helix to a **β**-strand upon an ATP binding under dilute conditions using circular dichroism (CD) spectroscopy. In contrast, under molecular crowding conditions, the **α**-helical conformation was stable even upon an ATP binding. These distinct conformational behaviors were observed to be independent of Na^+^ and Mg^2+^. Interestingly, CD measurements demonstrated that the spectra of a frayed duplex decreased with increasing of the RecG concentration both under dilute and molecular crowding conditions in the presence of ATP, suggesting that RecG unwound the frayed duplex. Our findings raise the possibility that the **α**-helix and **β**-strand forms of RecG are a preactive and an active structure with the helicase activity, respectively.

## 1. Introduction 

The double-stranded conformation of genomic DNA must be unwound to provide single-stranded DNA (ssDNA) intermediates required for DNA replication, recombination, and repair. The ssDNA intermediates can adopt various structures like junctions, G-quadruplex, and intramolecular triplex [1–3]. In cells, the unwinding of double-stranded DNA (dsDNA) is catalyzed by a class of ubiquitous enzymes termed DNA helicases [4]. Helicases disrupt one or more base pairs within the duplex DNA and then translocate vectorially to the next duplex region to repeat the process [5–9]. The helicase activity is cycled by the binding and hydrolysis of an NTP through a number of energetic (conformational) states that have different affinities for ssDNA and dsDNA [10]. 

The structure of helicases plays a critical role in their catalytic functions. Previously, it was reported that almost all helicases appear to function as oligomers (usually dimers or hexamers) [10]. Oligomerization provides multiple binding sites necessary for DNA or RNA target recognition, interaction with accessory proteins, and ATP binding [5, 6]. RecG is a well-characterized helicase from *Escherichia coli *that unwinds DNA junctions *in vitro*. Biochemical studies revealed that RecG is active as a monomer [11]. It catalyzes the interconversion of forks and junctions [1, 12, 13]. It is necessary in cellular processes such as DNA replication, recombination, and repair [5, 6]. The conversion of a replication fork into a Holliday junction requires the simultaneous unwinding of the leading and lagging strands followed by the reannealing of the two parental strands and the annealing of the two nascent strands. There is no information available on the relationship between the structural states of RecG and its function. Any study to determine the mechanism of RecG action must, therefore, be addressing the differences between structural states of inactive and active forms of RecG.

Nucleic acids possess an intrinsic structural polymorphism critical in nucleic acid-nucleic acid, nucleic acid-protein, and nucleic acid-drug interactions. The polymorphic properties are influenced not only by sequence but also by surrounding conditions. The structures adopted are especially influenced by ionic properties like ion concentration, charge, and size [14–16]. Moreover, living cells contain soluble and insoluble molecules such as proteins, nucleic acids, saccharides, lipids, and metabolites that can alter the stabilities of canonical and noncanonical nucleic acid structures [17]. The total concentration of biomolecules reaches 400 g L^−1^ in cells, leading to what is referred to as molecular crowding [17, 18]. In the crowded intracellular environment, water activity decreases and hydration is unfavorable. These crowded conditions stabilize the noncanonical DNA structures such as triplexes and G-quadruplexes, whereas they destabilize the duplex form [19, 20]. Elucidation of the interactions between helicases and DNA substrates with various structures is a very important step in understanding the mechanism through which helicases bind and unwind dsDNA. In this study, the structure and function of RecG were investigated under diluted and crowded conditions shedding light on how the structural properties of RecG correlate with activity.

## 2. Materials and Methods

### 2.1. DNA Sequences

DNA oligonucleotides of high performance liquid chromatography (HPLC) purification grade were purchased from Hokkaido System Science (see Table S1 in Supplementary Materials available online at doi:10.1155/2012/392039). Single-strand concentrations of the DNA sequences were determined by measuring absorbance at 260 nm at a high temperature using a Shimadzu 1700 spectrophotometer connected to a thermoprogrammer. Single-strand extinction coefficients were calculated from mononucleotide and dinucleotide data using the nearest neighbor approximation [21, 22].

### 2.2. Preparation of RecG

The gene encoding RecG was amplified using KOD-Plus DNA polymerase (Toyobo) with *E. coli* BL 21 genomic DNA as the template and the following primers: primer-S (5′-ggaattccatatgaaaggtcgcctgttagatg-3′) and primer-AS (5′-cccgctcgagtcatgcgttggagtaacgttc-3′). Restriction enzyme sites for digestion and ligation are underlined. The PCR products were inserted into the *Nde*I and *Xho*I sites of the pET-26b vector (Merck). RecG was expressed with a hexahistidine tag attached at the N-terminus.

RecG was expressed in *E. coli* strain Rosetta2 (DE3) at 28°C in LB medium supplemented with 30 mg L^−1^ kanamycin and 34 mg L^−1^ chloramphenicol. To induce RecG expression, isopropyl *β*-D-thiogalactopyranoside (IPTG) was added to a final concentration of 1 mM when the optical density of the cells reached approximately 0.6 at 600 nm. The culture was shaken overnight at 28°C. The procedure for purification RecG was as follows: cells were suspended in 20 mM Tris-HCl (pH 8.5), 100 mM NaCl, and membranes were disrupted with a sonicator. The soluble fraction was loaded onto a HiTrap HP column (GE Healthcare). RecG was purified over a HisTrap HP column using buffer A (20 mM Tris (pH 8.0) containing 100 mM NaCl) and buffer B (20 mM Tris (pH 8.0) containing 2 M NaCl) and then a HiLoad 26/60 Superdex 200-pg column (GE Healthcare) using buffer A (20 mM Tris (pH 8.0) containing 100 mM NaCl and 5 mM imidazole) and buffer B (20 mM Tris (pH 8.0) containing 500 mM NaCl and 1 M imidazole). Protein purity was confirmed by SDS-PAGE.

### 2.3. Circular Dichroism Measurements

Circular dichroism (CD) experiments were performed on a J-820 spectropolarimeter (Jasco) at 4°C and 37°C in a 0.1-cm path length cuvette. Samples of 5 *μ*M RecG were prepared in 30 mM MES (pH 7.0) and 0.5 mM Na_2_EDTA containing 100 mM NaCl or 100 mM NaCl and 1 mM MgCl_2_ or 5 mM MgCl_2_, with and without 1 mM ATP and 0 wt% or 40 wt% poly(ethylene glycol) with an average molecular mass of 200 (PEG 200). The CD spectra shown are the average of at least three scans from 200 to 350 nm. The temperature of the cell holder was regulated by a temperature controller (PTC-348, Jasco), and the cuvette-holding chamber was flushed with a constant stream of dry N_2_ gas to avoid condensation of water on the cuvette exterior. CD melting curves of RecG were recorded at 222 nm in the presence of 30 mM MES (pH 7.0) and 0.5 mM Na_2_EDTA containing 100 mM NaCl or 100 mM NaCl and 1 mM MgCl_2_ or 5 mM MgCl_2_ and 0 wt% or 40 wt% PEG 200. To analyze the functional activity of RecG, 1 *μ*M frayed duplex DNA was titrated with successive additions of 50 nM RecG and 0.1 mM ATP. Samples were prepared in the presence of 30 mM MES (pH 7.0) and 0.5 mM Na_2_EDTA containing 100 mM NaCl or 100 mM NaCl and 1 mM MgCl_2_ or 5 mM MgCl_2_. Before measurement, the frayed duplex DNA was heated to 95°C, gently cooled at a rate of 0.5°C min^−1^, and incubated at 4°C overnight.

### 2.4. UV Melting Analysis

UV absorbance was measured with the Shimadzu spectrophotometer equipped with the temperature controller. Melting curves of DNA structures were obtained by measuring the UV absorbance at 260 nm. Samples were prepared in 30 mM MES (pH 7.0) and 0.5 mM Na_2_EDTA containing 100 mM NaCl or 100 mM NaCl and 1 mM MgCl_2_ or 5 mM MgCl_2_. Before measurement, the samples were heated to 95°C, gently cooled at a rate of 0.5°C min^−1^, and incubated at 4°C overnight. Measurement was performed using a 1-cm path length cuvette. The melting temperature (*T*_*m*_) values for DNA structures were obtained from the UV melting curves as described previously [21, 22]. The heating rate was 0.5°C min^−1^.

## 3. Results and Discussion

### 3.1. Structures of RecG under Dilute Conditions with and without ATP

**Figure 1:**
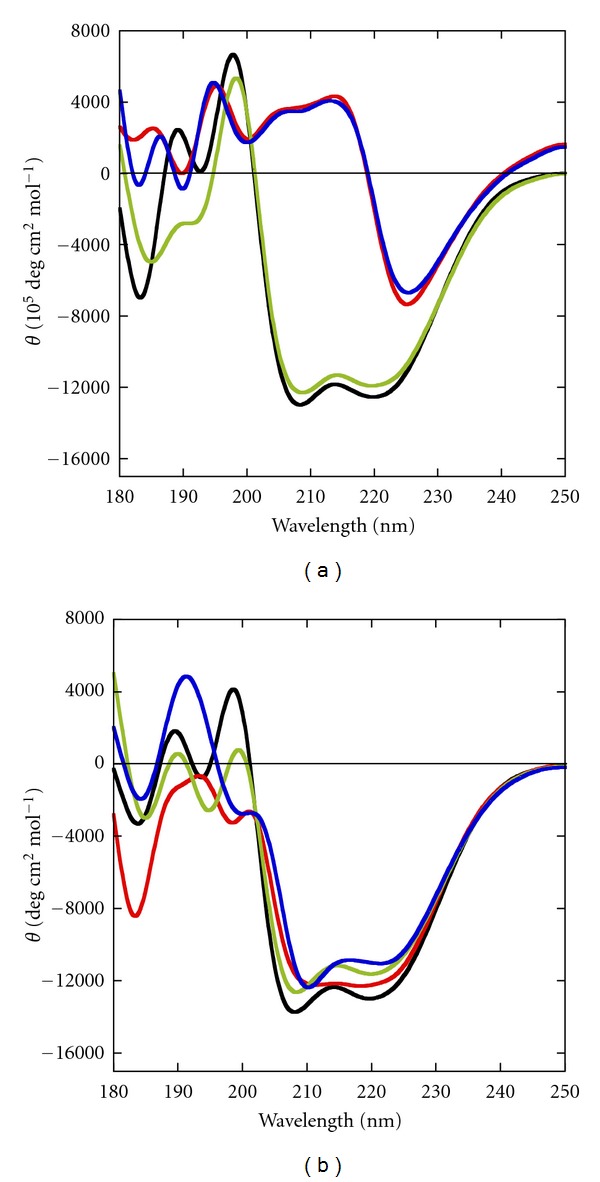
CD spectra of 5 *μ*M RecG. Measurements were carried out in 30 mM MES buffer (pH 7.0) containing 100 mM Na^+^ and 0.5 mM Na_2_EDTA (a) at 0 wt% PEG 200 without ATP at 4°C (black) and 37°C (green), or with 1 mM ATP at 4°C (red) and 37°C (blue), and (b) at 40 wt% PEG 200 without ATP at 4°C (black) and 37°C (green), or with 1 mM ATP at 4°C (red) and 37°C (blue), respectively.

Firstly, we used CD spectroscopy to study the structural states of RecG with and without ATP in the presence of different cations (Na^+^, Mg^2+^, or both Na^+^ and Mg^2+^) at 0 wt% and 40 wt% PEG 200, a neutral cosolute to study systematically the effects of molecular crowding. RecG was expressed in *E. coli* and purified by using ion exchange chromatography and affinity chromatography followed by dialysis.  Figure 1(a) shows CD spectra at 4°C or 37°C of 5 *μ*M RecG in the presence of 100 mM Na^+^ with or without ATP at 0 wt% PEG 200. The CD spectra without ATP displayed a positive peak at 198 nm and negative peaks at 208 nm and 220 nm, which are characteristic of an *α*-helix [23]. In the presence of 1 mM ATP, the CD spectra of RecG had a positive peak at 195 nm and a negative peak at 225 nm. These CD signatures are in agreement with formation of a *β*-strand structure [23]. 

The crystal structure of *Thermotoga maritime* RecG reveals three structural domains [24] (Figure S1). The largest domain (domain 1) is at N-terminus, which forms a long *α*-helix and a *β*-strand [25]. Domains 2 and 3 are referred to as helicase domains. The C-terminal residues of the protein extend from the end of domain 3 and cross-back to contact domain 1, forming a hook that wraps around the extended *α*-helix, which provides a nucleotide binding site [24]. Recently, the structure of *E. coli *RecG was modeled based on the coordinates of *Thermotoga maritime *RecG usingSwiss-Pdb Viewer [26]. Apart from the missing N-terminal sequences that form a separate fold in the *Thermotoga maritime *RecG, the *E. coli *RecG structure is essentially identical [27]. Morikawa suggested that the insertion before the helicase core in the RecG sequence compared to the *Thermotoga maritime *RecG contributes to the specific recognition of the branched DNA structure by *E. coli* RecG [28]. The *β*-strand in the insertion domain recognizes the junction through a stacking interaction with several aromatic residues in RecG [24] (Figure S1). Thus, the *α*-helix and the *β*-strand structures observed by the CD measurements likely correspond to the ATP binding domain and the DNA junction recognition structure, respectively.

We also measured the CD spectra of RecG as a function of ATP concentration from 0 mM to 1 mM under physiologically relevant ionic conditions (100 mM NaCl and 1 mM MgCl_2_) at 37°C (Figure S2a). The CD spectra of RecG without ATP displayed a positive peak at 196 nm and negative peaks at 210 nm and 222 nm that remained unchanged at ATP concentrations below 0.17 mM. At ATP concentrations from 0.34 mM to 1 mM, the CD spectra gradually shifted to a positive peak at 216 nm and a negative peak at 228 nm. Figures S2b and S1c show the plots of molar ellipticity at 210 nm and 225 nm, respectively, obtained from CD spectra versus increasing ATP concentration. The appearance of biphasic curves at 210 nm and 225 nm further confirms the structural transition. The difference of the transition points at 210 nm and at 225 nm might indicate that the dependency on ATP is different between the *α*-helix and the *β*-strand conformations. These CD results clearly indicate that the *α*-helix to *β*-strand transition in RecG depends on the ATP concentration.

### 3.2. Structures of RecG under Molecular Crowding Conditions with and without ATP

We also explored the effect of ATP binding on RecG under similar ionic conditions and a molecular crowding condition of 40 wt% PEG 200 (Figure 1(b)). CD spectra displayed positive peaks at 190 nm and 198 nm and negative peaks at 208 nm and 220 nm in the absence of ATP. In contrast to the spectra under the diluted conditions, no significant differences were observed in the CD spectra in the presence of 40% PEG 200 even after the addition of ATP, indicating that *α*-helix structure was dominant. In 100 mM NaCl and 1 mM MgCl_2_ (Figures S3a and S3b) or 5 mM MgCl_2_ (Figures S3c and S3d) at 0 wt% or 40 wt% PEG 200, we observed that RecG without ATP folded into *α*-helical conformation, whereas it was converted into the *β*-strand conformation after the addition of ATP. This result is identical to the result obtained with 100 mM Na^+^ only, indicating that under dilute conditions the binding of ATP to RecG regulates the structural transition from an *α*-helix to *β*-strand independent on the nature of cation. On the other hand, the cell-mimicking molecular crowding condition favored the folding of RecG into the *α*-helical conformation even in the presence of ATP. Previous studies have shown that molecular crowders can stabilize the native state of a protein [29, 30], promote a oligomerization [31, 32], shift an open-closed equilibrium toward a closed form as a substrate-binding state [17], and affect a folding rate of protein [33–35]. Protein-folding variants are proposed to have key roles in a number of pathophysiological processes [36–40]. In view of published results that support the hypothesis that molecular crowding conditions are more relevant to the conditions in cells than dilute conditions, the ATP-independence of the RecG folding may have biological significance.

### 3.3. Thermal Stability of RecG under Dilute Conditions with and without ATP

To investigate in detail the structural changes induced in RecG upon ATP binding, we explored the thermal stability of RecG using CD melting at 222 nm. Normalized CD melting curves of 5 *μ*M RecG in the presence of 100 mM Na^+^ (Figure S4a), 100 mM Na^+^ and 1 mM Mg^2+^ (Figure S4b), or 5 mM Mg^2+^ (Figure S4c) in the absence and presence of 1 mM ATP were recorded. A single melting transition was observed in all the conditions. The estimated values of melting temperature *T*_1/2_ (the temperature at which 50% of a protein sample is denatured) are given in Table S2. Addition of ATP stabilized the RecG by 3°C, 3.5°C and 1°C in the presence of 100 mM Na^+^, 100 mM Na^+^ and 1 mM Mg^2+^, or 5 mM Mg^2+^, respectively. Close internal packing of the backbone atoms in *β*-strand structures of RecG optimizes van der Waals interactions and minimizes energetically unfavorable hydrophobic interactions between nonpolar protein groups and water molecules in the environment [41]. Collectively, these factors help to reduce the net free energy of the *β*-strand and thereby increase its stability. Our results suggest that RecG adopts its active functional structure after ATP binding to facilitate the process of unwinding of nucleic acid substrate, because ATP bindings play an important role in the functional activity of various proteins [42–45]. 

### 3.4. Thermal Stability of RecG under Molecular Crowding Conditions with and without ATP

In the absence of ATP, RecG was stabilized by 2°C, 1.0°C, and 2.5°C in the presence of 100 mM Na^+^, 100 mM Na^+^ and 1 mM Mg^2+^, or 5 mM Mg^2+^ respectively at 40 wt% PEG 200 in comparison to 0 wt% PEG 200. Furthermore, addition of ATP thermally stabilized the RecG at 40 wt% PEG 200, and RecG was maximally stable (*T*_1/2_ = 50°C) at 40 wt% PEG 200 in the presence of ATP. Folded proteins usually have hydrophobic cores and charged or polar side-chains occupy the solvent-exposed surface. Minimizing the number of hydrophobic side chains exposed to water is an important driving force behind the folding process [46]. Formation of intramolecular hydrogen bonds provides another important contribution to protein stability [47]. The strength of hydrogen bonds depends on their environment, thus hydrogen bonds enveloped in a hydrophobic core contribute more than hydrogen-bonds exposed to the aqueous environment [48]. Under molecular crowding conditions, low water activity may induce the interdomain rearrangement in RecG to allow formation of the *α*-helix containing a hydrophobic core. The *α*-helix form was stabilized by 2°C (*T*_1/2_ = 50°C) at 40 wt% PEG 200 than at 0 wt% PEG 200 (*T*_1/2_ = 48°C) in the presence of 5 mM Mg^2+^.

### 3.5. Thermal Stability of Frayed Duplex under Dilute and Molecular Crowding Conditions

**Figure 2:**
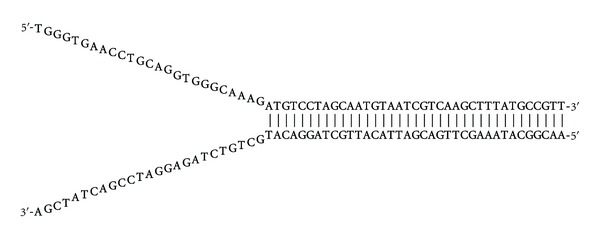
A nucleotide target sequence of frayed duplex.

We designed a 62-mer DNA oligonucleotide to form a frayed DNA duplex containing noncomplementary arms and a stem region (Figure 2). This arrangement of bases within the target duplex mimics that of a replication fork. The single-stranded non-complementary arms should facilitate the binding of RecG. The enzyme should then translocate to the dsDNA to allow the strand separation with a 3′–5′ polarity. The ssDNA extension was introduced at the 5′ end to stimulate the activity of RecG on the strand opposite to that used for the primary contact of RecG.

We first measured the stability of frayed duplex by recording the UV melting of 1 *μ*M DNA duplex in the presence of 100 mM Na^+^ (Figure S5a), 100 mM Na^+^ and 1 mM Mg^2+^ (Figure S5b), and 5 mM Mg^2+^ (Figure S5c) at 0 wt% or 40 wt% PEG 200. We observed two transitions for the thermal melting of the frayed duplex. The lower temperature transition could be due to the intramolecular interaction of the bases constituting the arm region of the frayed duplex. The higher-temperature transition could be due to the melting of the stem region. In the absence of PEG 200, the mid-points of the higher-temperature transitions were 70°C, 71°C, and 72°C in the presence of 100 mM Na^+^, 100 mM Na^+^ and 1 mM Mg^2+^, and 5 mM Mg^2+^, respectively. In 40 wt% PEG 200, the higher-temperature transitions in the presence of 100 mM Na^+^, 100 mM Na^+^ and 1 mM Mg^2+^, and 5 mM Mg^2+^ were 65°C, 66°C, and 66°C, respectively. To confirm the higher melting transition due to stem region, we have also recorded the stability of stem region of frayed duplex by recording the UV melting of 1 *μ*M DNA duplex in the presence of 100 mM Na^+^ (Figure S5a), 100 mM Na^+^ and 1 mM Mg^2+^ (Figure S5b), and 5 mM Mg^2+^ (Figure S5c) at 0 wt% or 40 wt% PEG 200. We observed single transition for the thermal melting of the stem region of frayed duplex which confirms that upper transition in frayed duplex is due to the intermolecular structure (Figure S6). In the absence of PEG 200, the mid-points of the higher-temperature transitions were 74°C, 74.5°C, and 74.5°C in the presence of 100 mM Na^+^, 100 mM Na^+^ and 1 mM Mg^2+^, and 5 mM Mg^2+^, respectively. In 40 wt% PEG 200, the higher-temperature transitions in the presence of 100 mM Na^+^, 100 mM Na^+^ and 1 mM Mg^2+^, and 5 mM Mg^2+^ were 74°C, 67°C and 66°C, respectively. The decrease in the *T*_1/2_ values under molecular crowding conditions is in good agreement with previous reports of DNA duplex stabilities in molecular crowders [49]. 

### 3.6. Functional Activity of RecG under Dilute Conditions

**Figure 3:**
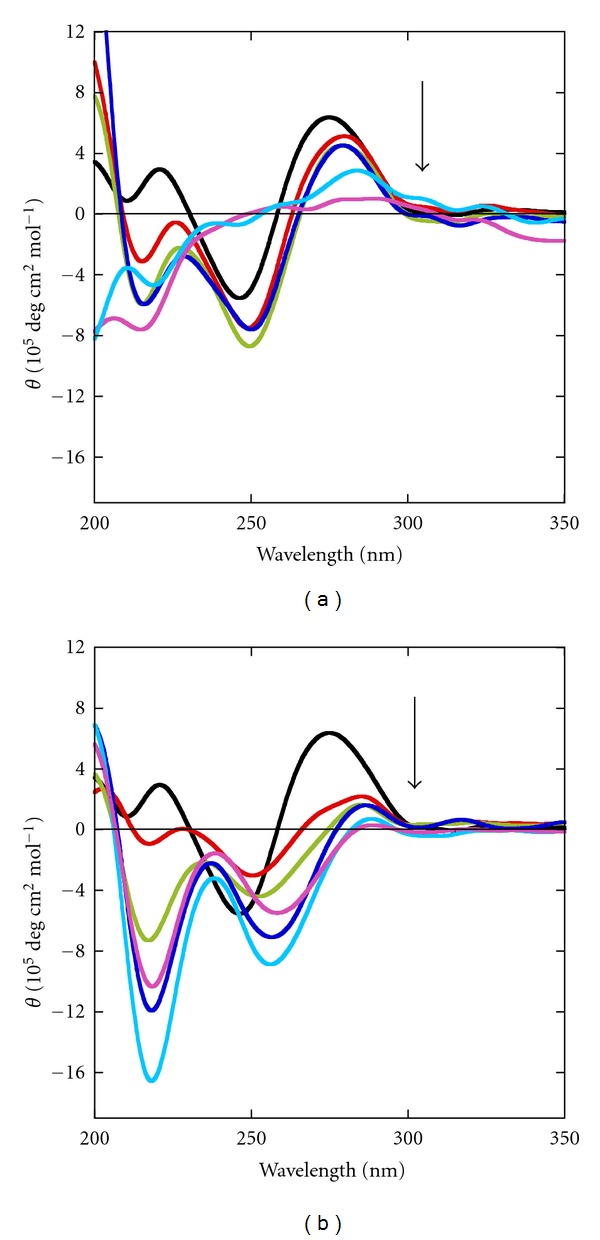
CD spectra of 1 *μ*M DNA frayed duplex. Measurements were carried out at 37°C in 30 mM MES buffer (pH 7.0) containing 100 mM Na^+^, 0.5 mM Na_2_EDTA, RecG (0 nM) and ATP (0 mM) (black), RecG (50 nM) and ATP (0.1 mM) (red), RecG (100 nM) and ATP (0.2 mM ) (green), RecG (150 nM) and ATP (0.3 mM, blue), RecG (200 nM) and ATP (0.4 mM, cyan), and RecG (250 nM) and ATP (0.5 mM) (pink), at (a) 0 wt% PEG 200 and (b) 40 wt% PEG 200.

The CD spectrum of the DNA in the presence of 100 mM Na^+^ without PEG 200 was characterized by a positive peak at 275 nm and negative peaks at 211 nm and 247 nm, a spectrum typical of the B-form conformation [50] (Figure 3). We then added RecG and ATP to the DNA. At 50 nM RecG and 0.1 mM ATP, we observed a decrease in intensity of the positive peak at 275 nm and a red shift such that negative peaks were located at 215 nm and 250 nm (Figure 3(a)). There was no signal from RecG due to the low concentration. Spectra were recorded after successive additions of RecG and ATP, and after each addition we observed a decrease in intensity of the positive peak. At 250 nM RecG and 0.5 mM ATP, there was complete loss in original B-form conformation of the DNA, indicating unwinding of the duplex. Previous report described the role of RecG in the processing of stalled replication forks, and acted by reversing the fork past the damage to create a four-way junction that allows template switching and lesion bypass [24]. It has also been reported that RecG unwinds both the leading and lagging strand duplex arms of a three-way junction and the unwinding of these arms was found to be coordinated [24]. In our study, as frayed duplex contains only single-stranded long overhangs at both the terminus, therefore, we propose that RecG should bind to the frayed duplex (as it mimics the replication fork) and should convert the duplex into single strands. Therefore, to confirm the same we have recorded the CD spectra of single stands constituting the frayed duplex under dilute and molecular crowding conditions (Figure S7). The CD spectrum of each single strand of frayed duplex in the presence of 100 mM Na^+^, 100 mM Na^+^ and 1 mM Mg^2+^, and 5 mM Mg^2+^ with and without PEG 200 was characterized by a positive peak at 274 nm and negative peaks at 244 nm. These CD signatures indicate that each single strand of the frayed duplex folds into typical of the B-form conformation due to the formation of intramolecular structure. To better understand the structures of frayed duplex after unwinding by RecG, secondary structures of single strands were predicted using M-fold [51, 52]. The monomers of frayed duplex showed 3 short complementary stems with a varying number of bases in loops along with short dangling ends at opposite terminus in each strand (Figure S8). When CD spectra were recorded after successive additions of RecG and ATP, and after each addition, we observed a decrease in intensity of the positive peak (Figure 3 and Figure S9). At 250 nM RecG and 0.5 mM ATP, there was complete loss in original B-form conformation of the DNA, indicating unwinding of the duplex into single strands solely due to the enzymatic activity of RecG in the presence of ATP. Earlier studies indicated that the substrate specificity of RecG was critically dependent on the concentrations of ATP and MgCl_2_, and under certain conditions, RecG preferentially unwound three strand junctions of DNA [1, 11]. Here, we observed functional activity of RecG in the presence of Na^+^ and absence of Mg^2+^.

Next, we recorded the CD spectra in the presence of 100 mM Na^+^ and 1 mM Mg^2+^ (Figure S9a) and in 100 mM Na^+^ and 5 mM Mg^2+^ (Figure S9b) without PEG 200. We observed an overall decrease in CD ellipticity at 273 nm that shifted to 283 nm after the successive additions of RecG and ATP, although the extent of decrease of the positive peak was observed to be less than that observed without Mg^2+^. These results clearly indicate that the functional activity of RecG was not dependent on the nature of the cations. A recent paper showed that the conformational change of the DNA-binding domain of hel308 helicase, which is a member of the same superfamily-2 helicase family as RecG, is coordinated by the ATP binding [53]. As we observed a structural transition from an *α*-helix to *β*-strand upon ATP binding under dilute conditions (Figure 1(a)) and functional activity under the same conditions, we propose that the *β*-strand structure is the conformation of RecG that unwinds our DNA substrate under dilute conditions. The *β*-strand structure could function as a “helix opener” by actively disrupting base pairs of a frayed DNA duplex, and this may be the basis of recognition of DNA junctions. 

### 3.7. Functional Activity of RecG under Molecular Crowding Conditions

To evaluate the effect of molecular crowding, we repeated our analysis of the activity of RecG in solution containing 40 wt% PEG 200. The CD spectra of the DNA were measured in 100 mM Na^+^ (Figure 3(b)), 100 mM Na^+^ and 1 mM Mg^2+^ (Figure S9c), and 5 mM Mg^2+^ (Figure S9d) in 40 wt% PEG 200. Of note, the conformation of the frayed duplex is different in 40 wt% PEG 200 than in 0 wt% PEG 200, as mentioned above stability of the frayed duplex was found to be lower under molecular crowding conditions than under dilute conditions. In 100 mM Na^+^ in the presence of the crowding agent, the positive peak at 275 nm shifted to 288 nm after the successive addition of 50 nM RecG and 0.1 mM ATP. This indicates the catalytic activity of RecG under molecular crowding conditions is different from that under the dilute conditions.

Similar decreases in intensities of the positive peak at 275 nm and the negative peaks at 221 nm and 247 nm were observed when RecG and ATP were added to the frayed duplex in the presence of 100 mM Na^+^ and either 1 mM Mg^2+^ or 5 mM Mg^2+^ and 40 wt% PEG 200 (Figures S9c and S9d). These CD results clearly indicate that RecG unwound the frayed duplex both under dilute and molecular crowding condition independent on the nature of cations, although it is quite possible that the extent of unwinding of frayed duplex and catalytic efficiency of RecG may depend on the nature of cation.

### 3.8. Possible Relationship between the Structure and the Function of RecG

In the absence of crowder and in the presence of ATP, RecG was converted from an *α*-helix form to a *β*-strand form. Under the crowding conditions, on the other hand, RecG remained in *α*-helix form even when ATP was added. The ATP binding site of RecG is in domains 2 and 3 which both have *α*-helical conformations [25]. It has been reported that the SecA helicase, which has homologous structure and function to RecG undergoes a transition to an *α*-helix form upon ATP binding [54]. Therefore, the *α*-helix form of RecG is likely essential for ATP binding. The conformational change to *β*-strand form observed in this study may suggest a transition to the active DNA-binding form of RecG. Based on our results, we propose that the *β*-strand form observed under diluted conditions is the active structure for binding and unwinding of DNA, whereas the *α*-helix form observed under the crowded conditions is the preactive structure that stably binds ATP. The different unwinding activities observed may be due to the interdomain flexibility of RecG, which allows substantial conformational changes imparting the ability to recognize more than one DNA structure. Furthermore, our results can provide significant information to explore and design the small ligands regulating the RecG helicase activity with a molecular crowding condition.

## 4. Conclusion

The data presented in this study emphasize some important points. Firstly, the secondary structures of RecG were different with and without a molecular crowding agent, PEG 200. The structural transition from *α*-helix to *β*-strand observed under the dilute conditions depended solely on the binding of ATP and not on the nature of cations present in solution. The molecular crowding conditions favored the folding of RecG bound to ATP into an *α*-helical structure. This may be a consequence of hydration. Secondly, the frayed duplex was unwound by RecG under dilute and molecular crowding conditions, indicating that the *β*-strand conformation may be the active structure of RecG, with the *α*-helix form the pre-active structure of RecG. Molecular crowding conditions played a critical role in the functional structure of RecG, and our data suggest the reason for the observed differences in the unwinding activity *in vivo* and *in vitro*. We are currently working to determine the binding stoichiometry of ATP with RecG and to quantify functional activity of RecG under dilute and molecular crowding conditions on various DNA substrates.
